# A new group of synthetic phenolic-containing amphiphilic molecules for multipurpose applications: Physico-chemical characterization and cell-toxicity study

**DOI:** 10.1038/s41598-018-19336-8

**Published:** 2018-01-16

**Authors:** Sampson Anankanbil, Bianca Pérez, Iva Fernandes, Katarzyna Magdalena Widzisz, Zegao Wang, Nuno Mateus, Zheng Guo

**Affiliations:** 1 Department of Engineering, Faculty of Science and Technology, Aarhus University, 8000 Aarhus, Denmark; 2 REQUIMTE/LAQV, Department of Chemistry and Biochemistry, Faculty of Sciences, University of Porto, 4169-007 Porto, Portugal; 3 Interdisplinary Nanoscience Center, Aarhus University, 8000 Aarhus, Denmark

## Abstract

Nine synthetic amphiphilic phenolic lipids, varied in phenolic moiety (caffeoyl/dimethylcaffeoyl) and fatty acid chain lengths (8–18) were characterized by differential scanning calorimetry (DSC), temperature-ramp Fourier transform infra-red spectroscopy (FT-IR) and atomic force microscopy (AFM). FT-IR and DSC results revealed that the physical state and lateral packing of synthetic molecules were largely governed by fatty acyls. The critical micelle concentrations (CMC) of synthetic lipids was in the range of 0.1 mM to 2.5 mM, affording generation of stable oil-in-water emulsions; as evidenced by the creaming index (<5%) of emulsions stabilized by compounds **C12**‒**C16**, and **C12a**‒**C16a** after 7 days’ storage. AFM analysis revealed that compound **C14** formed stable double-layers films of 5.2 nm and 6.7 nm. Application studies showed that formulations stabilized by synthesized compounds containing 30% fish oil had superior physical and oxidative stability compared to formulations containing commercial emulsifiers or their mixtures with phenolic acids. Moreover, the synthetic compounds were non-toxic against *in vitro* transformed keratinocytes from histologically normal skin and Caco-2 cell lines. This study demonstrates the relevance of using a natural hydroxycarboxylic acid as a flexible linker between natural antioxidants, glycerol and fatty acids to generate multifunctional amphiphiles with potential applications in food, pharmaceutical and cosmetic industry.

## Introduction

Fats and oils, carbohydrates, carboxylic acids and phenolic acids have been demonstrated to be valuable raw materials for the development of functional materials for various applications^1–9^. However, the aforementioned raw materials are either hydrophilic (e.g. carboxylic acids, carbohydrates and phenolic acids) or hydrophobic (fats, oils and fatty acids) which limit their application in foods, drugs and cosmetics, where amphiphilic molecules are needed^1–9^.

**Figure 1:**
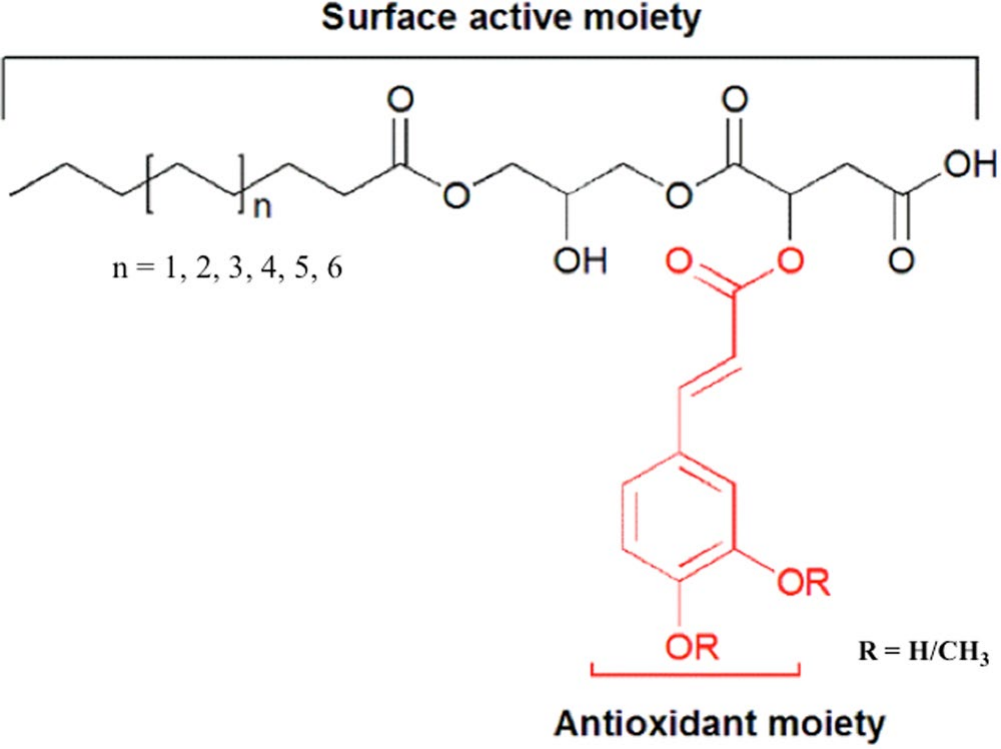
General structure of malic acid esters of monoglycerides. Fatty acid chain length is represented by ‘**n**’. Compounds **12a**–**16a** contain caffeic acid as phenolic moiety while compounds **8**–**18** contain 3,4-dimethoxycinnamic acid.

In a previous work, the rational design of a 2-step synthetic approach permitted the assembling of natural building blocks into a single amphiphilic agent to yield antioxidant emulsifiers (Fig. 1)^10^. Malic acid was used as flexible linker between natural phenolic acids and monoacylglycerols. The resulting compounds differed from any phenolipids previously reported due to the presence of a hydroxycarboxylic acid moiety, which potentially enable interactions with macromolecules^11^, and thereby further stabilize droplets of lipophilic ingredients enclosed in the hydrophobic core. Therefore, the compounds are expected to be used as high capacity delivery cargos (e.g. ~70% fish oil) for lipophilic ingredients due to their multifunctional properties (surface activity and antioxidant property), and ability to interact with macromolecules. In addition, due to structural similarities to other compounds in the literature^1–9^, the synthesized amphiphiles could potentially find applications not only in food but also in cosmetics, or as nanocarriers. For instance, longer alkyl chain derivatives of this series of compounds could be applied in cosmetic formulations as protective skin barriers^5,6^.

Thus, the aim of this work was to further characterize malic acid esters of monoglycerides through multiple physicochemical techniques including Differential Scanning Calorimetry (DSC), temperature-ramp Fourier Transform Infrared spectroscopy (FTIR), and Atomic Force Microscopy (AFM) and confirm their great potential for industry applications. Cytotoxicity assays of the synthesized compounds on a normal cell line established from the skin of a 62-years old Caucasian man were also performed. Moreover, the functionality of the compounds was investigated by measuring the ability of the amphiphilic lipids to stabilize 30% oil-in-water emulsions. Furthermore, the antioxidant potency of the amphiphilic compounds in emulsions was determined using thiobarbituric acid reactive substances (TBARS).

## Results and Discussion

### Synthesis and characterization

Six compounds (**C8**, **C10**, **C12**, **C14**, **C16** and **C18**) based on 3,4-dimethoxy-cinnamic acid and three compounds (**C12a**, **C14a** and **C16a**) based on caffeic acid (Fig. 1) were synthesized and fully characterized. Synthesis was carried out following a procedure previously described in the literature^10^. Briefly, phenolic acids were respectively made react with malic acid to form a phenoleoyl-malic acid anhydride, which was then coupled to monoglycerides by a ring opening mechanism^4^. The structures of all synthesized compounds were accurately identified by use of MS and NMR (^1^H, ^13^C), and further characterized by use of DSC, FT-IR and atomic force microscopy (AFM). In addition, the ability of the compounds to function as emulsifiers was tested by measuring creaming index. The ability of the compounds to inhibit lipid oxidation in emulsions was further determined. Moreover, the cytotoxicity of the compounds was evaluated using *in vitro* transformed keratinocytes from histologically normal skin (HaCAT) and human colorectal adenocarcinoma (Caco-2).

### Thermal analysis by use of Differential Scanning Calorimetry

The amphiphilic lipids were characterized by means of DSC to determine their melting transition temperatures. This is relevant for applications as emulsifiers^12^, skin lipids^5,6^, or nanocarriers^9^. No clear melting transition temperatures were observed for compounds **C10**‒**C16** when temperature scanning from was done −60 °C to 60 °C. On the other hand, a melting point of 38 °C was observed for compound **C18** (Figure S1). DSC results suggest that multi-acylation with bulky groups (in the form of phenolic and malic acids) of the glycerol backbone of monoglycerides frustrates the molecular packing of the resulting compounds leading to disorganized structures^6^. However, elongating the hydrocarbon chain to 18 carbons sufficiently increased the van der Waals interaction of the hydrocarbon chain to overcome the internal repulsions of the ester and carboxylic functional groups, and hence promoted a better organization of the molecules^5,6^ consequently leading to a melting point above the room temperature (See Table 1 for a summary of the physicochemical properties of the synthesized compounds).

**Table 1:** Physicochemical characterization of synthesized phenolic lipids.

Compound	Yield (%)	m.p* (°C)	Lipid packing mode	Vibrational modes (3000‒2800 cm^−1^ region)	
				vCH_2_ symmetric stretching	vCH_2_ asymmetric stretching
**C8**	19	liquid	Hexagonal	2854	2923
**C10**	41	liquid	Hexagonal	2854	2921
**C12**	65	liquid	Hexagonal	2853	2923
**C14**	61	liquid	Hexagonal	2852	2921
**C16**	58	liquid	Hexagonal	2852	2922
**C18**	43	38	Orthorhombic	2848	2916
**C12a**	63	liquid	Hexagonal	2852	2921
**C14a**	58	liquid	Hexagonal	2852	2921
**C16a**	55	liquid	Hexagonal	2852	2921

*Except for compound C18, all other compounds are liquid at room temperature.

### Determination of lateral organization of alkyl chains of amphiphiles

**Figure 2:**
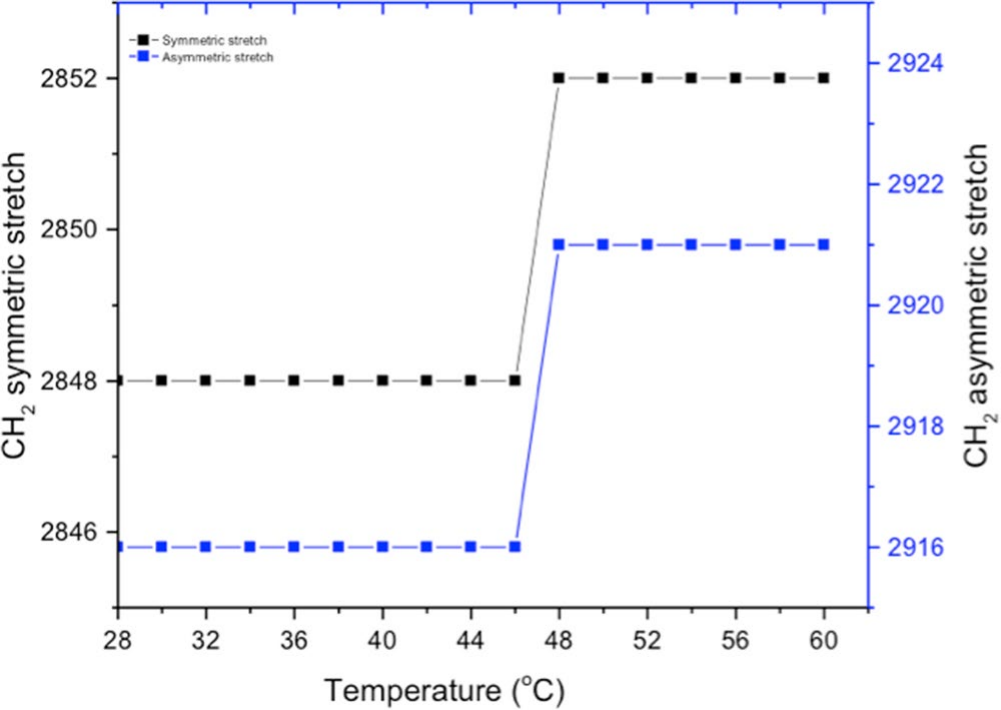
Phase transition of the CH2 symmetric and asymmetric vibration bands of compound C18 as a function of temperature. Spectra were recorded in the temperature range of 28‒60 °C.

Even though DSC provides valuable information about the thermal transitions of compounds, little is gained about their molecular organizations^9^. FT-IR is a powerful tool to acquire detailed information about the conformations and molecular orientations of alky side chains of lipids^13^. The aim of the FT-IR study was to establish further structure-activity relationships for the amphiphilic lipids as this knowledge is relevant for applications of the compounds as emulsifiers^9^, skin lipids^5,6^ or as agents for drug delivery^9^. The CH_2_ bending bands (∼720‒725 cm^−1^) and CH_2_ symmetric stretching bands (∼2920 cm^−1^ and 2850 cm^−1^) were of particular interest as these regions provide information regarding lipid chain organization. The CH_2_ rocking region (720–740 cm^−1^) of synthesized compounds **C8, C10, C12, C14, C16, C12a, C14a** and **C16a** at 28 °C displayed a single peak indicating that these compounds present hexagonal packing (Table 1 summarizes the packing modes of the various amphiphilic lipids), which suggest less organized lipid structures. However, compound **C18** showed a doublet at 720 and 740 cm^−1^, which indicates orthorhombic packing, a more organized conformation of the molecules(Figure S2 shows the rocking region of **C10** and **C18** as an example). The orthorhombic packing behavior displayed by compound **C18** suggests that long hydrocarbon chains (>16) are required to generate enough van der Waals interactions to compensate for the intermolecular repulsions from the ester and carboxylic functional groups in the molecules. This finding corroborates previous reports from other authors^5,14,15^, and shows that the lengths of hydrocarbon chains affect molecular organization of amphiphilic lipids. In addition, compound **C18** displayed peaks at lower frequencies in the CH_2_ symmetric and asymmetric stretching regions (2848 cm^−1^ and 2916 cm^−1^, respectively) compared to compounds **C10** to **C16** (2852-2854 cm^−1^ and 2921–2923 cm^−1^, respectively) suggesting a more organized lipid chain conformation. Frequency shifts in symmetric and asymmetric stretching of hydrocarbon chains can also be used to predict conformational changes under thermal stress. High frequencies indicate disordered chain packing modes while low frequencies indicate that the hydrocarbon chains are in the well-ordered *all-trans* conformation^16^. Hence, the FT-IR results showed that the alkyl chains of **C18** were in the *all-trans* conformation at 28 °C. This highly organized conformation is observed up to 46 °C degrees where the lipids chains become disordered (Fig. 2). In other words, compound **C18** is able to maintain its molecular organization until 46 °C where disorderliness sets in. This result implies longer derivatives (>C22:0) of malic acid esters of monoglycerides can potentially be excellent skin barrier material and can therefore find value in cosmetic formulations, where tight molecular organizations are essential^5,6,17^.

On the contrary, the rest of the compounds (**C8**, **C10**, **C12**, **C14**, **C16**, **C12a**, **C14a** and **C16a**) displayed disorganized lipids chains at room temperature with peaks above 2852 cm^−1^ and 2921 cm^−1^ in the FTIR spectra. The latter is in agreement with DSC analysis where only **C18** displayed a melting point above 25 °C and the rest of the compounds being liquid at this temperature. The similarities in terms of the molecular organization and physical state of compounds **C8**‒**C16** to other amphiphilic compounds^6,12^ reported suggest that they could find application as emulsifiers in foods^12^ and cosmetics^6^. Previous studies showed that amphiphilic lipids with short alkyl side chains are more digestible in the gastrointestinal tract compared to amphiphiles with longer alkanyl side chains^18^. Therefore, **C8**‒**C16** derivatives could favor more rapid digestion by gastrointestinal tract (GIT) lipases and could be applied for the encapsulation and hence rapid release of omega-3 oils in the GIT.

### Surface activity of amphiphiles

**Figure 3:**
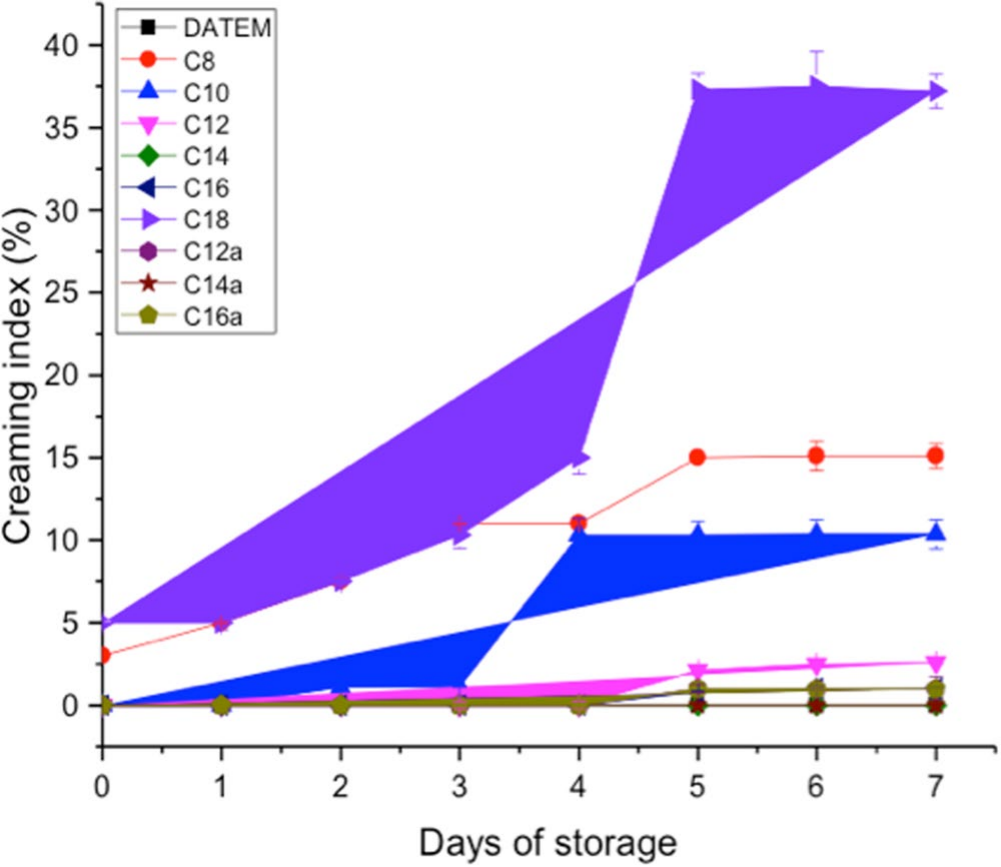
Creaming index of emulsions stabilized by phenolic lipids and commercial DATEM as a function of days of storage at 5 °C.

The application of a surfactant depends on its surface active properties. One of the analytical methods to determine the surface activity of emulsifiers is by measuring emulsion stability^19^ (creaming index, CI) as function of time of storage. In this work, the CI of oil-in-water emulsions were determined as a function of time and correlated to CMC (Figure S3 in supporting information) of the amphiphilic lipids. CI was generally low during early days of storage (day 0 to day 3), increased gradually to a maximum and then remained stable (Fig. 3). Emulsions stabilized by compounds **C12**, **C12a**, **C14**, **C14a**, **C16** and **C16a** had comparable CI as commercial DATEM, and were thermodynamically stable throughout the period of storage (0% CI). However, creaming was highest in emulsions stabilized by compound **C18** (37.2%) followed by compound **C8** (15.1%) and then compound **C10** (10.35%) after 7 days of storage, suggesting that there was no correlation between the length of the hydrocarbon chain and the CI of the different phenolic emulsifiers.

It is interesting to note that certain ranges of CMC values favored emulsion stability. Compounds **C12**, **C12a**, **C14**, **C14a**, **C16** and **C16a**, which formed the most stable emulsions, had CMC values in the range of 0.1 mM to 2.5 mM. As depicted in Fig. S3, the change of in CMC is a function of carbon chain length of fatty acyl moiety. CMC was found to decrease with increasing length of the hydrocarbon chain of amphiphilic lipids. This suggests that a decrease in hydrophobicity of amphiphilic lipids with the same head group is accompanied by an increase in CMC, a trend similar to results from other works^20,21^. However, structural features such as size, conformation, and configuration of surfactants also play important roles in determining CMC of surfactants^22^.

In the broader interpretation of these results, it could be explained that compound **C18** being more hydrophobic had a very low CMC (0.01 mM) and consequently, a poor surface activity as evidenced by CI measurements. On the contrary, **C8** and **C10** were very hydrophilic and presented high CMC values (10 mM and 5 mM, respectively). Hence, **C8** and **C10** will preferentially reside in the aqueous phase of emulsions and that explains the high CI in emulsions stabilized by **C8** and **C10**. In summary, the CI and CMC data suggest that the compounds could be categorized into three main groups namely, the highly hydrophilic group (**C8** and **C10**) with high CMC and good surface activity, the surface active group (**C12**‒**C16**) with intermediate CMC, and the highly hydrophobic group (**C18**) with very low CMC and very poor surface activity.

### Determination of antioxidant properties of amphiphiles

Compounds **C12a**‒**C16a** being caffeic acid derivatives are expected to be potent antioxidants and could be used as encapsulating agents for oils or drugs sensitive to oxidation. Therefore, an investigation of the ability of the synthesized compounds to inhibit lipid peroxidation in fish oil enriched emulsions was carried out for the most surface active compounds (**C12a**, **C14a** and **C16a)**. In addition, an emulsion stabilized with commercial DATEM was used as a control while another emulsion stabilized with commercial DATEM but with added caffeic acid was used for comparison.

**Figure 4:**
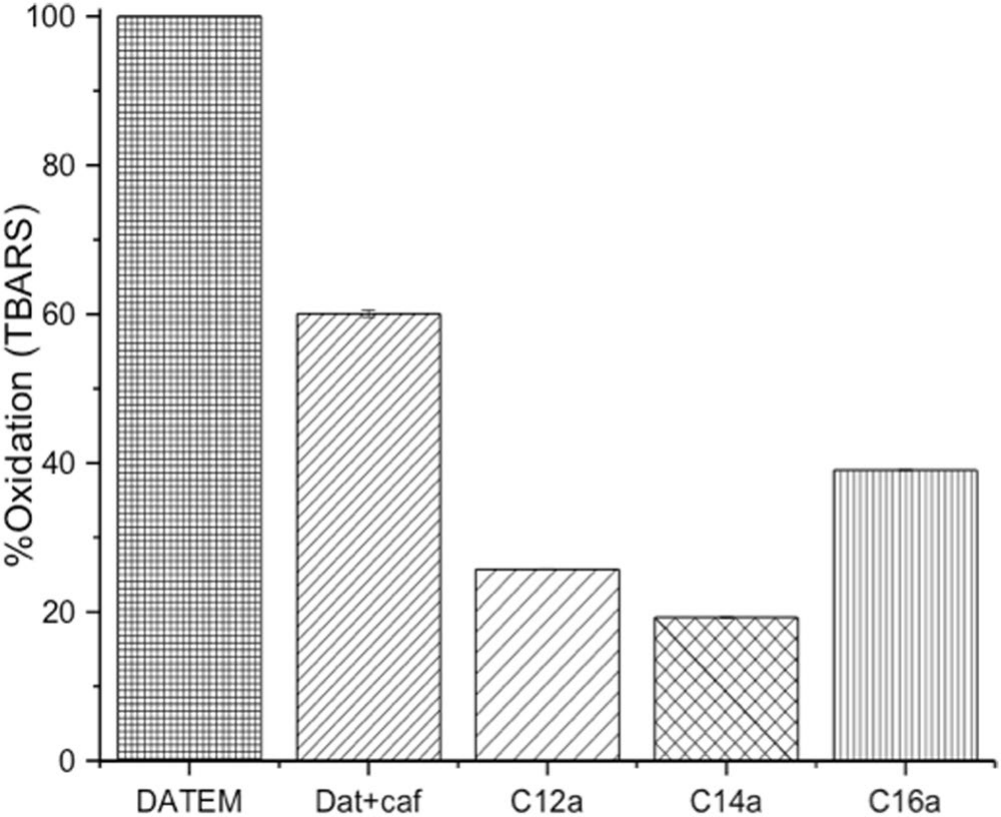
Relative oxidation in emulsions (as TBARS) stabilized by phenolic lipids **C12a**, **C14a** and **C16a** compared with oxidation in emulsions stabilized by commercial DATEM without antioxidant activity, and commercial datem with caffeic acid (Dat + caf), after 1 week of storage.

As displayed on Fig. 4, all the tested compounds inhibited lipid oxidation in emulsions. For instance, compared with a commercial DATEM with no antioxidant activity, there was only 19.26 ± 0.05% oxidation in emulsions prepared with **C14a** after 7 days of storage. However, oxidations were significantly (p < 0.05) higher in emulsions prepared with compounds **C12a** and **C16a** compared to emulsions stabilized with **C14a**. In emulsions, transition metals and other agents which promote lipid oxidation, are present in the aqueous phase, and come into close contact with lipids on the surface of oil droplets^22^. Thus, compound **C14a**, being an excellent surface active agent as demonstrated by the creaming stability, is expected to be highly concentrated at the oil-water interface and offer a better protection against lipid oxidation.

The degree of oxidation observed in emulsions stabilized by the new compounds are significantly (p < 0.05) lower than the 20.8 ± 2.7% reported for 20% omega-3 oil-in-water emulsions stabilized by dodecenyl succinic anhydride modified alginate^2^. Furthermore, lipid oxidation was high in emulsions prepared with mixtures of commercial DATEM and caffeic acid compared to emulsions prepared with the phenolic emulsifiers **C12a**, **C14a** and **C16a**. This could be due to a higher concentration of the hydrophilic caffeic acid in the aqueous phase and consequently, a lower concentration at the oil-water interface of the emulsions stabilized with commercial DATEM. Moreover, a high-density of phenolic acids at oil-water interface sets a more effective barrier to prevent penetration of preformed free radicals into the core of encapsulated oil.

In other words, this result provides evidence that this group of synthetic molecules elaborately integrates multi-function into a single molecule by bringing otherwise water-soluble phenolic acids into the interface where lipid oxidation occurs. This agrees with a previous report^22^ that shows significantly higher lipid peroxides in emulsions stabilized by Tween 20 with added erythorbic acid than in emulsions stabilized with the surface active compound erythorbyl laurate. To the best of our knowledge, the amphiphilic molecules here presented, are the first series of molecules which are sufficiently rich in hydrophilic, hydrophobic and antioxidant moieties that can find value in a wide range of applications.

### Atomic Force Microscopy of monolayers

**Figure 5:**
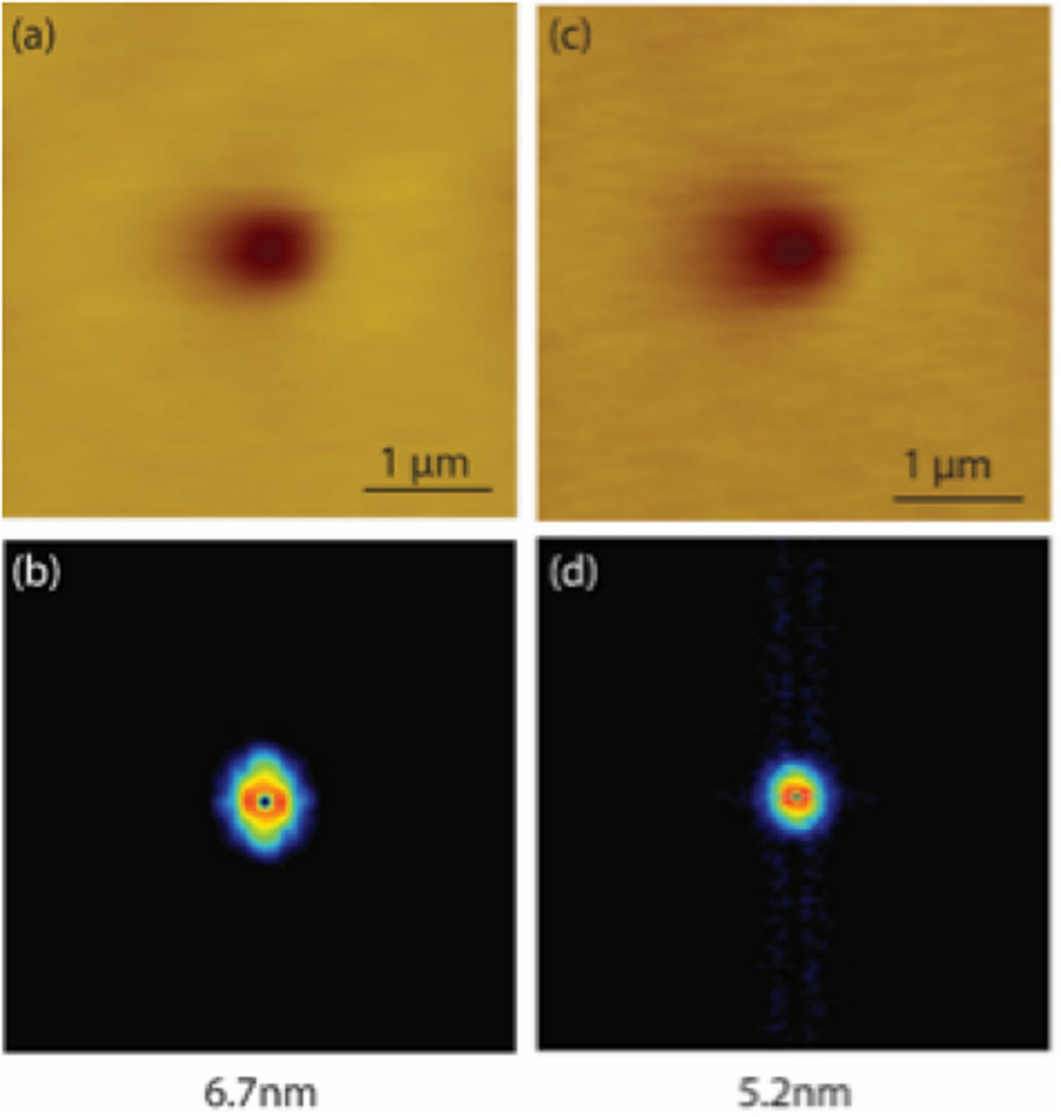
AFM images (**a** and **c**) of compound **C14** at different regions on mica. (**b** and **d**) are the corresponding FFTT results of **C14**.

The results from CI determinations and antioxidant activity assay showed that **C14** was the optimal molecule for delivery of high loads of omega-3 oils. **C14** was therefore chosen for further studies using AFM (Fig. 5 and Figure S4). The Langmuir isotherm of compound **C14** showed that monolayer collapse occurred at surface pressures of 39 mN/m. Therefore, a pressure in the liquid condensed phase (~22 mN/m) was selected to transfer monolayers of **C14** onto a solid hydrophilic mica substrate for AFM studies. AFM images showed that compound **C14** was prone to form stable double-layer films (Film thickness = 5.2 nm and 6.7 nm). Nevertheless, monolayers of 2.3 nm of thickness were also observed. The molecular length of compound **C14** from the methyl group of the fatty acid chain to the hydroxyl moiety at the para-position of caffeic acid is approximately 3.4 nm, when the fatty acid chain is fully extended. Therefore, it can be inferred that at P = 22 mN/m mixtures of monolayers and double-layer are present. Since the film thickness found was 2.3 nm but the theoretical length of a molecule of **C14** is 3.4 nm, it can be concluded that the molecules are tilted relative to the mica substrate^13,23^ The significance of the ability of **C14** molecules to form monolayers means the hydrophobic core could be loaded with lipophilic ingredients such as omega-3 oils making **C14** an excellent delivery vehicle for bioactive compounds^9^. In addition, a film thickness of 6.7 nm, which is about two molecular lengths of compound **C14**, indicates the presence of double layer^9^. This result demonstrated that compound **C14** has the potential to form liposome structure useful for encapsulation of hydrophilic compounds of medicinal interest.

Fast Fourier transform filtering (FFTT) was applied to the AFM images of **C14** to evaluate possible patterns in periodicity and directions of molecular alignments^6^. However, contrary to results observed for glycerol monobehenate^6^ and lignoceric acid^24^, FFTT result (Fig. 5b) revealed no periodicity patterns for sample **C14** which demonstrates that the observed stripes in Fig. 5d for AFM images of compound **C14** are a result of the imaging process rather than a property of the system.

### Toxicity evaluation of amphiphilic lipids

**Figure 6:**
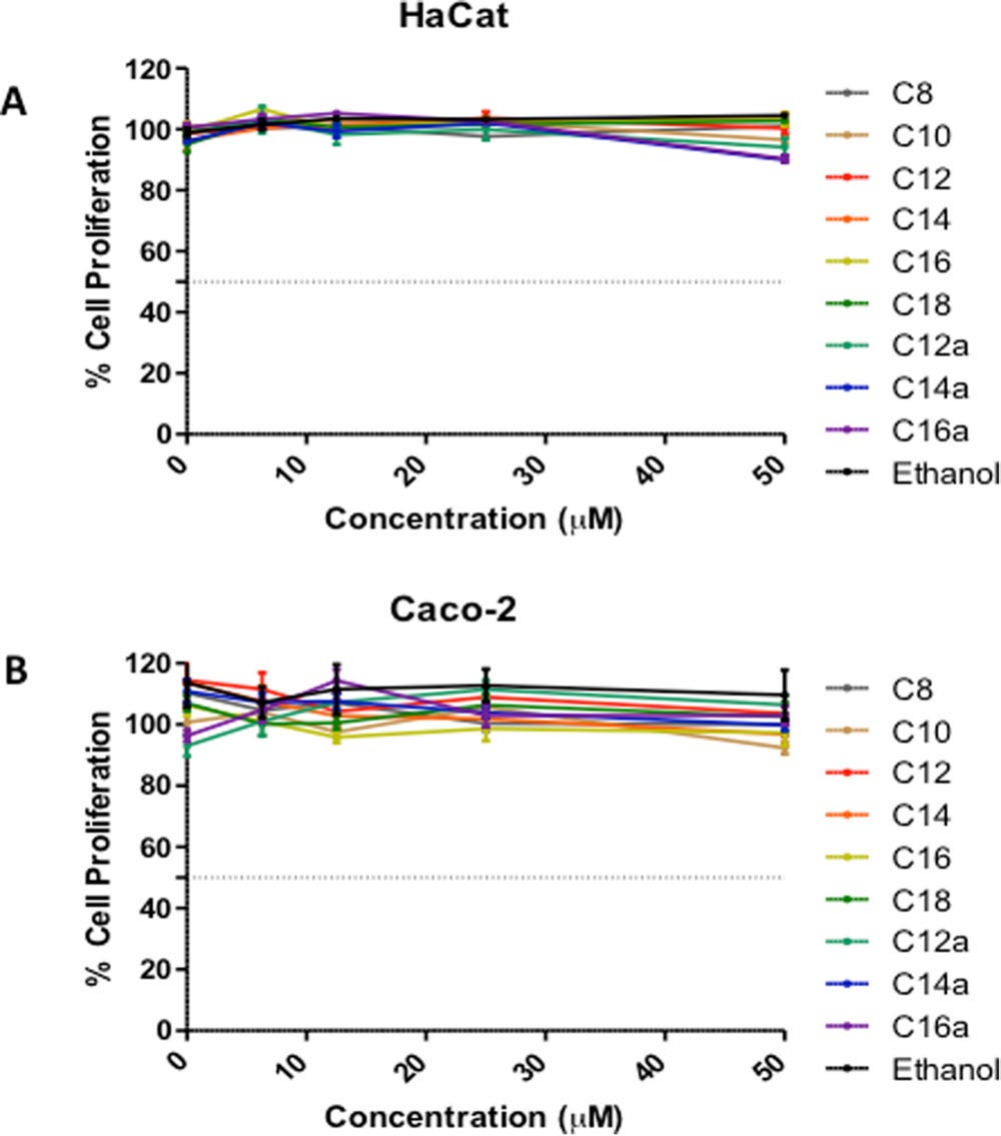
Effect of increasing concentrations of synthesized amphiphilic lipids on proliferation of HaCat. (**A**) and Caco-2 (**B**) cells.

Since consumers prefer ingredients from natural sources, efforts are continually being made to replace synthetic ingredients with their natural counterparts^25^. Thus, to evaluate the hypothesis that joining compounds from natural source will lead to non-toxic emulsifiers, cytotoxicity assays of the synthesized compounds using *in vitro* HaCat and Caco-2 cell lines were performed. The results show that cell proliferation was 100% with non-noticeable cell death when cultured in increasing concentrations of solutions of the new amphiphiles (from 0μM to 50μM) (Fig. 6A,B). These results support the view that assembling natural molecules into a single multifunctional compound may lead to non-toxic agents.

## Conclusions

A new series of phenolic-containing amphiphilic compounds were synthesized from renewable raw materials. The compounds were physicochemically characterized by use of DSC, Temp-ramp FTIR and AFM. FT-IR studies revealed that the compounds adopt a hexagonal packing with the exception of compound **C18** which exists in the tight orthorhombic packing mode. The orthorhombic packing mode of **C18** suggests that it could be applied as encapsulation matrix^25^ for oxidation sensitive drugs and that longer derivatives of malic acid esters of monoglycerides can find value as skin protective barrier material for cosmetic formulations similar to other long chain amphiphiles reported^5,6^. Therefore synthesis of ultra-long chain derivatives of these compounds could provide novel functional skin lipids.

In addition, the surface-active properties of the compounds were confirmed by their ability to form stable fish oil-in-water emulsions as measured by creaming index. Compounds **C12**‒**C16** & **C12a**‒**C16a** had excellent surface activity with very stable emulsions (CI < 5%) even after 30 days of storage. Moreover, compounds **C12a**, **C14a** and **C16a** significantly (p < 0.05) inhibited lipid oxidation in emulsions compared to mixtures of commercial DATEM and caffeic acid because of their high surface activity, and preferential location of the phenolic moiety at the oil-water interface where lipid oxidation occurs. However, lipid oxidations were significantly higher (p < 0.05) in emulsions prepared with compounds **C12a** (25.72 ± 0.03% and **C16a** (39.04 ± 0.02%) compared to emulsions stabilized by compound **C14a** (19.26 ± 0.05%) suggesting that **C14a** was highly concentrated at the interface of the oil-in-water emulsions. Compared to other antioxidant emulsifiers reported, compounds **C12a**, **C14a** and **C16a** are comparable or even superior at protecting omega-3 oils against oxidation^2^. Therefore, compounds **C12a**‒**C16a** represent new materials for protecting oxidative sensitive fish oils against chemical deterioration.

Atomic force microscopy of Langmuir-Blodgett films of the optimal surface active and antioxidant compound (**C14**) on mica substrate revealed the formation of stable double-layer films (Film thicknesses of 5.2 nm and 6.7 nm), and monolayer films of 2.3 nm thickness. It can be inferred that the ability of these compounds to form stable monolayer films in addition to their superior surface activity and antioxidant properties mean that they can be applied for the delivery of lipophilic ingredients with high load capacity. This is an improvement compared to conventional emulsifiers or stabilizers for delivery of bioactives^2,22,27^. Moreover, since the compound is capable of forming stable double layers of 5.2 nm and 6.7 nm, it can potentially form liposome structures and find application in the encapsulation of hydrophilic compounds prompt to oxidation.

Furthermore, cytotoxicity assays using HaCAT showed that the new multifunctional amphiphiles are non-toxic and could be used as ingredients for food, cosmetics or in pharmaceutical formulations. This is the first study to use natural a hydroxycarboxylic acid as a flexible linker between natural antioxidants, glycerol and fatty acids to generate a new series of multifunctional amphiphiles with potential applications in food, drug and cosmetic applications.

## Materials and Methods

Glycerol, caffeic acid, 3,4-dimethoxycinnamic acid, 2,2-diphenyl-1 picrylhydrazyl (DPPH), tricholoroacetic acid (TCA), thiobarbituric acid (TBA), sodium salicylate >99.5% (C_7_H_5_NaO_3_), pyrene, H_2_O_2,_ pyridine (anhydrous, >99%) and all other chemicals were purchased from Sigma-Aldrich (St. Louise, USA). 4,4-difluoro-5-(4-phenyl-1,3-butadienyl)-4-bora-3a,4a-diaza-*s*-indacene-3-undecanoic acid (C11-BODIPY^581/591^)) was obtained from Molecular probes (Leiden, The Netherlands), and Diacetyltartaric acid esters of mono- and di-glycerides (DATEM) was obtained as a gift from Dupont Nutrition and Health (Brabrand, Denmark). All water used was de-ionized water obtained from a Milli-Q (Millipore, MA) system. Structural elucidation of target compounds was done using a Bruker Maxis Impact electrospray ionization quadrupole time-of-flight mass spectrometer (ESI-QTOF-MS).^1^H and ^13^C NMR spectroscopy were carried out on a Bruker Avance III spectrometer at 400 MHz. FT-IR Spectra were recorded using an ATR-FTIR (PIKE, Madison, WI; Bruker, Ettlingen, Germany). TLC was used to monitor the progress of the reaction using a solvent mixture of diethylether:petroleum ether:acetic acid (85:15:1 v/v/v).

### General procedures for synthesis of amphiphilic lipids

#### Synthesis of monoglycerides

Monoglycerides of various fatty acid chain lengths (8, 10, 12, 14, 16 and 18) were synthesized using Novozyme 435 (*Candida antarctica* lipase B) in *t*-BuOH. A typical reaction consisted of 2 mmol of fatty acid, 24 mmol of glycerol, Novozym 435 (8% of total weight of equivalent amount of fatty acid) and 200 mg molecular sieves (3 Å, activated by heating up to 180 °C for 8 h) in 5 mL t-BuOH, a reaction time of 4 h and magnetic stirring at 360 rpm. The reactions mixtures were incubated in 50 ml glass jacketed reactors at a temperature of 40 °C for 30 min before the addition of the lipase.

After the reaction, the mixture was extracted with 50 mL DCM, washed three times with saturated Na_2_CO_3_ solution to remove the unreacted fatty acid, and then three times with a saturated NaCl solution. After removal of the trace amounts of water with anhydrous sodium sulfate, the organic phase was separated and the solvent was removed by rotatory evaporation under vacuum.

#### Synthesis of phenolic acids-hydroxycarboxylic acid adducts (compounds 1a & 1b)

Approximately 2 g of 3,4-dimethoxycinnamic acid or caffeic acid was placed in a 250 ml two-necked round bottom flask fitted with a condenser and the flask placed in an oil-bath at a constant temperature of 90 °C with mechanical agitation at 360 rpm. Thionyl chloride was gradually added from a dropping funnel through the open end of the flask over a period of 30 min and the reaction maintained for 2 h at 90 °C and the excess thionyl chloride distilled off. After cooling to room temperature malic acid (mole ratio of malic acid: phenolic chloride, 1:2) was added. The mixture was warmed to 70 °C for 4 h with stirring at 360 rpm. After cooling to room temperature, the resulting product thoroughly washed with diethylether to remove excess phenolic acid and dried under nitrogen to obtain a white powder (**Compound 1a:** *3,4-dimethoxy-cinnamoyl malic anhydride* and **Compound 1b**: *3,4-dihydroxy-cinnamoyl malic anhydride*). Though this procedure employs less environmentally friendly reagents and solvents, we are currently developing a greener alternative reaction employing biocatalysts.

Compound **1a**. White powder. HRMS: calculated for C_15_H_16_O_8_Na [M + Na] + :347.0737; found: 347.0745.

Compound **1b**. White powder. HRMS: calculated for C_13_H_12_O_8_Na [M + Na] + : 319.0424; found: 319.0487.

#### Synthesis and structural identification of phenolic emulsifiers

To a solution of the respective monoacylglycerol (1.78 g) in 5 ml anhydrous DMF, compound **1a** or compound **1b** (0.5 g) was added. The reaction mixture was cooled to 0 °C and 0.002 moles of dry pyridine was added. Stirring was continued at 360 rpm under nitrogen for 30 min, and then at room temperature for 16 h. After completion of the reaction, 2 N HCl was added at 0 °C with vigorous stirring and the mixture extracted with ethyl acetate. The organic phase was washed three times with brine, dried over anhydrous sodium sulfate, filtered and vacuum dried to yield the desired product. A total of 9 compounds were synthesized by this procedure. Six compounds (**C8**, **C10**, **C12**, **C14**, **C16** and **C18**) based on 3,4-dimethoxy-cinnamic acid and three compounds (**C12a**, **C14a** and **C16a**) based on caffeic acid (Fig. 1). Detailed spectroscopy data can be found elsewhere^10^.

### Physico-chemical characterization of synthesized compounds

#### Differential scanning calorimetry measurement

The thermal properties of the compounds were analyzed using Differential Scanning Calorimetry (DSC) instrument on Pyris 6 system (Perkin-Elmer Cetus, Norwalk, USA). Compounds were dried under low pressure over night, and then encapsulated in aluminum pans. The measurement was under an atmosphere of nitrogen with flow of 20 mL/min. The heating and cooling profile was: 1) initial temperature 0 °C; 2) ramp 10 °C /min to 60 °C; 3) isothermal for 5 min; 4) ramp 10 °C /min to −60 °C; 5) isothermal for 5 min; and 6) ramp 10 °C /min to 60 °C. The DSC scans were evaluated by using MicroCal Origin 9.0 software.

#### Temp-Ramp- Fourier transform infrared spectroscopy measurement

Fourier transform infrared spectroscopy (FT-IR) was applied to determine the molecular organization of the amphiphilic lipids. Spectra were recorded using an ATR-FTIR (PIKE, Madison, WI; Bruker, Ettlingen, Germany). The synthesized compounds were dried under low pressure over night, and then pressed onto a ZnSe ATR crystal mounted in a trough plate. The ATR crystal was coupled with an Auto Pro Temperature Controller (Pike Technologies, Madison, WI) for gradual heating of the crystal from 30 °C to 60 °C. Spectra were collected with a spectral resolution of 4 cm^−1^ with 8 scans over the range of 3500–650 cm^−1^. The FTIR spectra were analyzed by using MicroCal Origin 9.0 software.

#### Determination of critical micelle concentration of emulsifiers

Critical Micelle Concentration (CMC) values of the synthetic compounds were determined by pyrene fluorescence method^28^ using a Varian Cary Eclipse Fluorescence spectrometer (Agilent Technology, California, USA). The respective solutions were prepared using water previously saturated with pyrene (1 µM final concentration) in different concentrations (0.000001, 0.00001, 0.0001, 0.001, 0.01, 0.1, 1, 2.5, 5, 10, 15, 20 mM). Emission spectra of pyrene were obtained by exciting the samples at 334 nm. The fluorescence intensity ratio of *I*_1_*/I*_3_ (I_1_ = 373 nm, I_3_ = 383 nm) was plotted against sample solution concentration. The concentration at which the first break occurs was referred as the CMC value of the compound in water. Measurements were determined in triplicate.

#### Atomic Force Microscopy

Langmuir-Blodgett studies was carried out for compound **C14**, being the ultimate compound with both surface active and antioxidant properties, according to the method of Correa *et al*.^29^ with slight modications. Experiments were performed in aqueous solutions at neutral pH. Briefly, 20 µl of 2 mg/mL solution of **C14** in chloroform:methanol (9:1, v/v) was applied to the aqueous phase and allowed to evaporate for a period of 20 min. Thereafter, the barriers were compressed at a constant rate of ~9 Å/ (chain min) until film collapsed. After stable conditions were attained, deposition of the corresponding film was carried out onto a hydrophilic mica support at a pressure just before film collapsed. The deposited monolayers on mica were left to air dry overnight. AFM images were acquired at ambient conditions by air tapping mode using a silicon tip on a micro cantilever (Olympus Inc., Japan) with a spring constant of 26 N/m and resonant frequency of 300 kHz. All measurements were performed in the center of the sample. Analyses were done in duplicates.

### Formulation of oil-in-water delivery emulsions

The synthetic compounds were evaluated as emulsifiers for fish oil-in-water emulsions and compared to commercial DATEM. Emulsions consisting of 30.0% fish oil were prepared using 1.5 mL (0.1 M) of emulsifier solutions in phosphate buffer (pH 7.0). Coarse emulsions were first created by homogenization of lipid and aqueous phases (PRO250, PRO Scientific, Oxford, USA) at high speed for two minutes at room temperature. Further reduction particle size was achieved by use of a probe sonicator (Branson sonifier 250, Branson ultrasonics, Danbury, US) for a period of 3 min.

#### Creaming stability of emulsions

To examine their creaming stability, duplicate emulsions were transferred to clear, screw-capped 15 mL vials (diameter 1.8 cm) and stored at 5 °C. By aid of daily digital photographs, the phase separation in each emulsion was monitored over 7 days. The extent of creaming was characterized by the creaming index (CI) according to Equation (2), where H_A_ is the height of the lower, aqueous phase, and H_E_ is the total height of emulsion^30^).

1 $$ \% CI=\frac{{H}_{A}}{{H}_{E}}\times 100$$

#### Determination of lipid oxidation in emulsions

The ability of the new emulsifiers to inhibit lipid oxidation in emulsions was evaluated using Thiobarbituric acid-reactive species (TBARS) assay. TBARS were used to measure the formation of malondialdehyde, a major product of lipid oxidation^31^, in emulsions after one week of storage. A solution of TCA-TBA-HCl was prepared by mixing 15 g of TCA, 375 mg TBA, 1.76 ml 12 N HCl, and 82.9 ml water. Two millimeters of this solution was mixed with 20 µl of the emulsion sample previously in 1 ml of distilled water. Thereafter, the mixture was heated at 100 °C for 15 min, cooled to room temperature within 10 min under a running tap water, and centrifuged at 2000×*g* for 15 min^22^. TBA formed colored complexes with the secondary oxidation products, which were detected in a UV-visible spectrophotometer (Cary 50Bio, Varian, Australia) at 532 nm. The relative degree of oxidation in each emulsion was calculated with respect to the degree of oxidation in emulsions with commercial DATEM, according to Equation (3), where A_S_ is the absorbance of samples with added stabilizer, and A_0_ is the absorbance of samples with no added emulsifier. Measurements were taken on duplicate emulsion samples.

2 $$Oxidation\,( \% )=\frac{{A}_{S}}{{A}_{O}}\times 100$$

#### Statistical analysis

Data processing was performed in Microsoft Excel 2010. All measurements were conducted in duplicates/triplicates, and the results are reported as means ± standard deviations. One-way analysis of variance (one-way ANOVA) was performed using Microsoft Excel Analysis Toolpak (2010) to identify significant differences between groups (p < 0.05).

### Toxicity evaluation of amphiphilic lipids

#### Cell Culture

An aneuploid immortal keratinocyte cell line from adult human skin, HaCat was cultured in RPMI (Sigma, Madrid, Spain) medium supplemented with 10% heat-inactivated FBS and a human colorectal adenocarcinoma cell line, Caco-2 in minimum essential medium Eagle supplemented with 15% fetal bovine serum, 25 mM HEPES. Both media were added with 1% antibiotic/antimycotic solution (100 units mL^−1^ of penicillin, 100 μg mL^−1^ of streptomycin and 0.25 μg mL^−1^ of amphotericin B) (all from Sigma). Cells were kept at 37 °C in a humidified atmosphere with 5% CO_2_ and harvested by trypsinization (0.25% (w/v) trypsin-EDTA_4_Na) twice a week.^26^

#### Sulfo-rhodamine B assay

The effect of compounds on the growth of the human keratinocyte cell line (HaCat) and Caco-2 cells was assessed according to the procedure adopted by the US National Cancer Institute in the “*In vitro Anticancer Drug Discovery Screen”* that uses the protein-binding dye SRB to assess cell growth. HaCat and Caco-2 cells were first grown into 96-well plates at a cellular density of 1.5 × 10^5^ cell/mL in RPMI (Sigma, St. Louis, MO) medium and allowed to grow for 24 h. Thereafter, cells were incubated with a four-serial concentration of the compounds (6.3, 12.5, 25, 50 µM), for 48 h, with a maximal solvent concentration (ethanol) of 0.05%. TCA (50% solution) was then added and after 1 h at 4 °C, the plates were washed four times with deionized water and allowed to dry overnight. The wells were then stained with 0.4% solution of SRB for 30 min in the dark. The excess of staining solution was washed out with 1% acetic acid and the bound stain was solubilized with tris-buffer (10 mM, pH 8.0) and the absorbance measured at 492 nm in a microplate reader (Powerwave XS, Bio-Tek Instruments Inc.). Cytotoxicity was determined as percent survival, calculated by the number of treated (*T*) over the control (*C*) cells × 100% (% *T/C*).

## Electronic supplementary material

Supplementary Information
