# Supplementary material for: A new group of synthetic phenolic-containing amphiphilic molecules for multipurpose applications: Physico-chemical characterization and cell-toxicity study
Source: Sci Rep. 2018 Jan 16;8:832. doi: 10.1038/s41598-018-19336-8 (PMC5770433; doi:10.1038/s41598-018-19336-8)
Supplement: Supplementary file 1 — Supplementary Information [file 41598_2018_19336_MOESM1_ESM.pdf]

Supporting Information

A new group of synthetic phenolic-containing amphiphilic molecules for multipurpose applications: Physico-chemical characterization and cell-toxicity study

Sampson Anankanbil[1], Bianca Pérez[1], Iva Fernades[2], Katarzyna Magdalena Widzisz[1], Zegao Wang,[3] Nuno Mateus[2] and Zheng Guo[1*]*

[1]Department of Engineering, Faculty of Science and Technology, Aarhus University, 8000 Aarhus, Denmark;

[2]REQUIMTE/LAQV, Department of Chemistry and Biochemistry, Faculty of Sciences, University of Porto, 4169-007 Porto, Portugal.

[3]Interdisplinary Nanoscience Center, Aarhus University, 8000 Aarhus, Denmark;

* Corresponding authors: Professor Zheng Guo, Gustav Wieds vej 10, Aarhus 8000-DK, email: guo@eng.au.dk, phone: +4587155528 and Dr. Bianca Pérez, Gustav Wieds vej 10, Aarhus 8000-DK, email:bperez@eng.au.dk, phone: +4587155527.

TABLE OF CONTENT

DSC thermograms of amphiphilic lipids…………………………………………………….....3

FT-IR lateral packing modes of compounds C10 and C18 ……………………………………….3

Variation of Critical micelle concentration (CMC) as a function of alkyl chain length of amphiphilic lipids………..4

Three dimensional atomic force microscopy (AFM) image of compound **C14** on mica………………………………...4

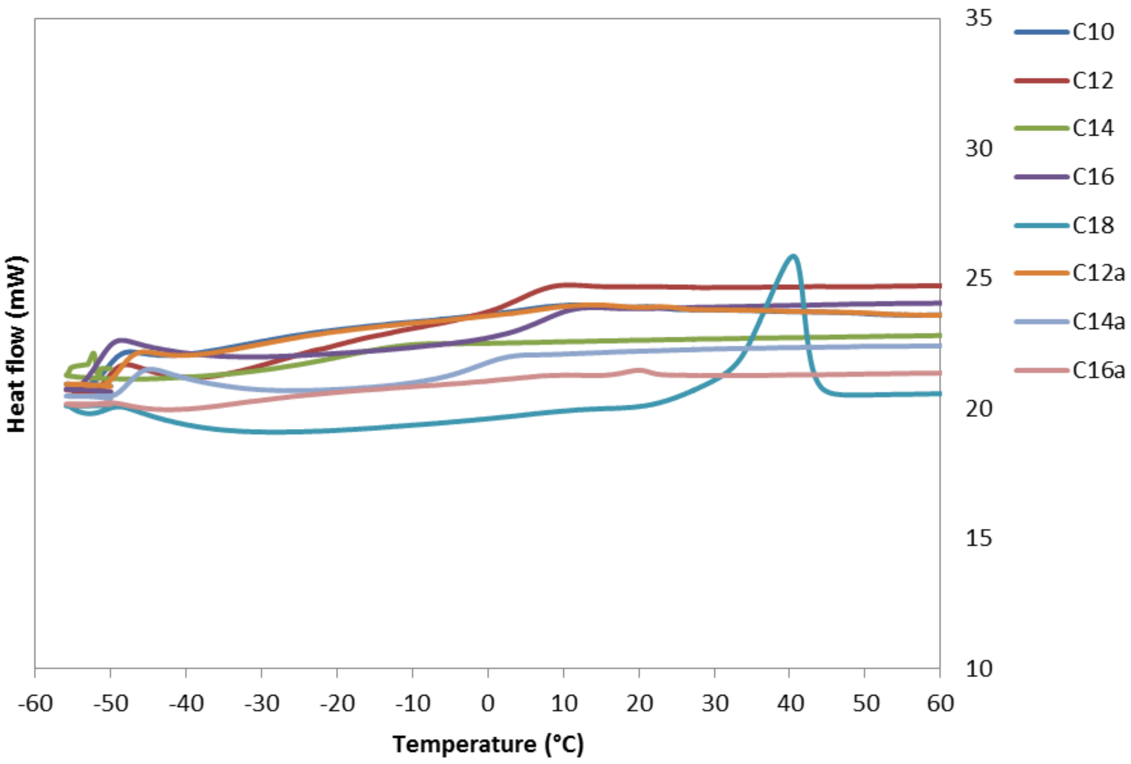

Figure S1. DSC thermograms of amphiphilic compounds **C10–C18** & **C12a–C16a**.

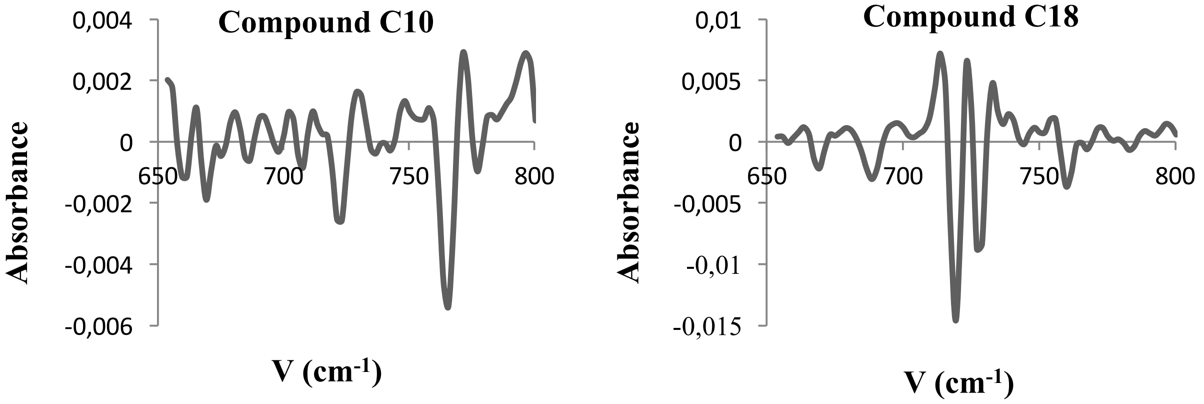

Figure S2. FTIR spectra of compound **C10** (hexagonal packing) and compound **C18** (orthorhombic packing) in the region of 650–800 cm^{-1} (Second derivative function using savitzky-Golay filter was applied). The peaks between 720 and 750 cm^{-1} indicate the strength of interchain interactions, which are stronger in **C18** than in **C10**.

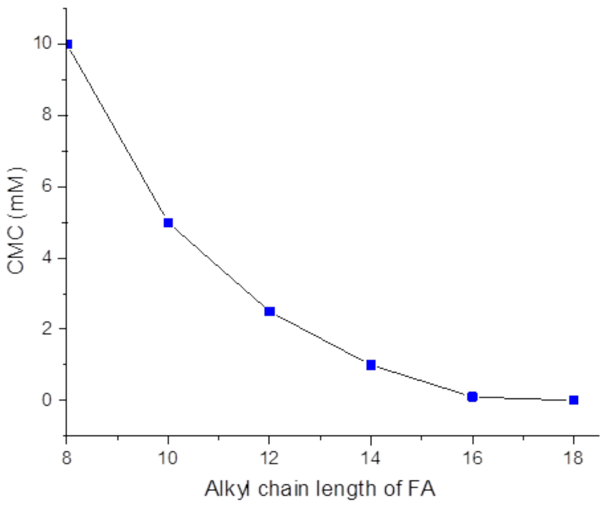

Figure S3. Variation of CMC with alkyl chain length of amphiphilic lipids

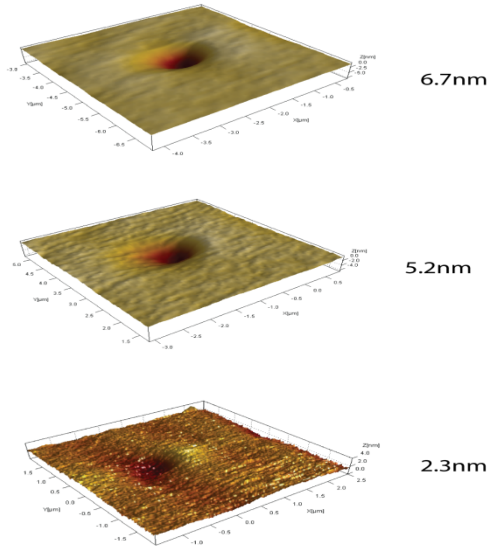

Figure S4. Three dimensional AFM images of compound **C14** deposited on mica.
